# Cost-Effectiveness Analysis of Three Leprosy Case Detection Methods in Northern Nigeria

**DOI:** 10.1371/journal.pntd.0001818

**Published:** 2012-09-20

**Authors:** Charles Ezenduka, Erik Post, Steven John, Abdulkarim Suraj, Abdulahi Namadi, Obinna Onwujekwe

**Affiliations:** 1 Health Policy Research Group, Department of Pharmacology and Therapeutics, College of Medicine, University of Nigeria, Enugu, Nigeria; 2 Department of Clinical Pharmacy and Pharmacy Management, Nnamdi Azikiwe University, Awka, Nigeria; 3 Royal Tropical Institute (KIT), Amsterdam, The Netherlands; 4 State Tuberculosis and Leprosy Control Programme (STBLCP), Yola, Nigeria; 5 State Tuberculosis and Leprosy Control Programme (STBLCP), Gombe, Nigeria; 6 Netherlands Leprosy Relief (NLR), Jos, Nigeria; 7 Department of Health Administration and Management, University of Nigeria, Enugu, Nigeria; Fondation raoul Follereau, France

## Abstract

**Background:**

Despite several leprosy control measures in Nigeria, child proportion and disability grade 2 cases remain high while new cases have not significantly reduced, suggesting continuous spread of the disease. Hence, there is the need to review detection methods to enhance identification of early cases for effective control and prevention of permanent disability. This study evaluated the cost-effectiveness of three leprosy case detection methods in Northern Nigeria to identify the most cost-effective approach for detection of leprosy.

**Methods:**

A cross-sectional study was carried out to evaluate the additional benefits of using several case detection methods in addition to routine practice in two north-eastern states of Nigeria. Primary and secondary data were collected from routine practice records and the Nigerian Tuberculosis and Leprosy Control Programme of 2009. The methods evaluated were Rapid Village Survey (RVS), Household Contact Examination (HCE) and Traditional Healers incentive method (TH). Effectiveness was measured as number of new leprosy cases detected and cost-effectiveness was expressed as cost per case detected. Costs were measured from both providers' and patients' perspectives. Additional costs and effects of each method were estimated by comparing each method against routine practise and expressed as incremental cost-effectiveness ratio (ICER). All costs were converted to the U.S. dollar at the 2010 exchange rate. Univariate sensitivity analysis was used to evaluate uncertainties around the ICER.

**Results:**

The ICER for HCE was $142 per additional case detected at all contact levels and it was the most cost-effective method. At ICER of $194 per additional case detected, THs method detected more cases at a lower cost than the RVS, which was not cost-effective at $313 per additional case detected. Sensitivity analysis showed that varying the proportion of shared costs and subsistent wage for valuing unpaid time did not significantly change the results.

**Conclusion:**

Complementing routine practice with household contact examination is the most cost-effective approach to identify new leprosy cases and we recommend that, depending on acceptability and feasibility, this intervention is introduced for improved case detection in Northern Nigeria.

## Author Summary

Reported increases in child proportions and disability grade 2 cases in Nigeria suggest that leprosy disease is still spreading in the country. This indicates the need to review case finding strategies to improve case detection for effective control of the disease. It was necessary that available methods be assessed for their value for money in view of limited resources. We evaluated the cost-effectiveness of three available leprosy case detection methods using data available from 2005 to 2011. We explored data to determine which strategy when implemented in addition to routine practice would detect most additional leprosy cases at a given cost (measured in U.S. dollars). Hence, cost-effectiveness was expressed as incremental cost-effectiveness ratio (ICER). Our findings show that at the rate of $142 per additional case detected, the household contact examination was the most costeffective strategy for detecting additional leprosy cases when implemented to complement routine practice.

## Introduction

Leprosy is a communicable disease caused by a bacillus, *Mycobacterium Leprae*, which can lead to permanent disability among sufferers with significant psychosocial and economic burden. The disease causes skin lesions and nerve damages which progress to deformities of the eyes, hands and feet [1]–[2]. These physical disabilities are the prominent features of the disease which impose stigmatisation and discrimination on the sufferers [3]. The negative impact of leprosy due to stigmatisation is more than most other infectious diseases, making sufferers with physical disability dread stigmatisation and discrimination by the society [4].

In recognition of the burden of leprosy the World Health Organisation (WHO) in 1991 set a goal to eliminate the disease by 2000, defined as reducing the prevalence to less than one case per 10,000 populations [5]. Key targets of strategy for the reduction of burden of leprosy are the timely detection of new cases and prompt treatment with Multi Drug Therapy (MDT), which is the standard treatment for leprosy [6]. Since the progression of leprosy prognosis is insidious, taking an average of 2–5 years to manifest due to slow growth and multiplication of *M. Leprae*, early identification of the disease is very critical for effective control. This reduces both transmission of M. leprae and prevents disability.

Nigeria achieved WHO global leprosy elimination target of less than 1 case per 10,000 in 1998, but the country remains among those that still report relatively high number of registered leprosy cases [7]. According to the NTBLCP Annual Report 2009, implementation of MDT in the country as the strategic intervention to eliminate leprosy resulted to a rapid decline of registered cases from approximately 200,000 in 1989 to 6,906 in 2008 [7]. Disability Grade 2 (DG2) among the new cases were 14% while child proportion was 12%. The National Strategic Plan for Tuberculosis and Leprosy Control in Nigeria [8] is in line with the WHO's Enhancing Global Strategy for Further Reducing Disease Burden Due to Leprosy 2011–2015 [9]. The plan set the goal for reducing DG2 cases by 35% from 2010 figures by the end of 2015. Objectives include timely case finding and treatment, monitoring and limiting the progress of DG2 to not more than 5% annually. The plan recommends cost-effective approach to leprosy control interventions to ensure achievement of set goals. Several measures have been in place to control the disease in Nigeria, with support from international agencies such as the Netherlands Leprosy Relief (NLR) agency and other International Federation against Leprosy (ILEP) members. However the fact that indicators such as child cases remain high at 10% while DG2 stay at 14%, higher than target [7], [8], raises concern about hidden cases and continuous spread of the disease as well as the effectiveness of current detection methods in identifying early cases. This calls for the review of various detection methods to ensure more effective control of the disease in the region. Existing strategies include the routine/passive case detection (PCD), active case finding through the mini Leprosy Elimination Campaign (Mini- LEC) and contact examination.

Published studies suggest that effectiveness of different detection methods vary between settings and contact levels. For instance the effectiveness of HCE varies between countries and this tends to depend on leprosy endemicity and definition of ‘contact’. In high endemic communities in Indonesia, it was found that almost 80% of new cases could be defined as contacts [10]. In Orissa 72 additional cases could be detected through HCE of 400 index cases [11] in higher endemic areas, although in Bangladesh spatial clustering of new patients at household levels could not be clearly established [12], while in low endemic areas relatively higher proportions of new cases could be found among household contacts of index cases [13]. The RVS strategy has played important roles in India, Indonesia, China and Thailand [14], [15], [16], [17]. As part of the mini-LEC in Indonesia, it detected twice higher leprosy prevalence than routine programme activities [15]. It is known to detect cases early which mean shorter treatment for PB cases and less number of disabilities and leprosy reactions. Evidence of Traditional healers' contribution to leprosy case detection has also been documented [18]. They have gained the confidence of the community given their level of successes and affordable costs of treatment. Due to stigmatisation status of leprosy many suspected carriers would rather visit traditional healers for privacy than public health facilities or programmes. Hence, the need to collaborate with recognised practitioners to improve identification of leprosy cases in the community.

The objective of this study was to establish the cost-effectiveness of the three alternative leprosy case detection strategies in comparison to current practice with the aim to identify more efficient method for achieving the goals of the national programme in limiting child proportion and disability grade 2 cases.

## Methods

### Study area and population

The study was conducted in Adamawa and Gombe states, two neighbouring north-eastern states of Nigeria with a combined population of over 5.8 million people in 2009, based on the 2006 population projection. The people are subsistent farmers and nearly 50% of the population are below 15 years old. There are over 1905 health facilities which include 2 Federal Medical Centers, 1 dermatology hospital, (which serves as a Leprosy referral center in the area), 1 specialist hospital, 15 general hospitals, and over I100 primary health centers (PHCs). Only about 11% (195) of the health facilities are private while the rest are public. 127 facilities provide leprosy services in Adamawa state while the dermatology hospital serves as the referral center. In line with national guideline Leprosy services are combined with Tuberculosis as National Tuberculosis and Leprosy Control Programme (NTBLCP) established in 1988. Both services are supported by the Netherland Leprosy Relief agency.

Although leprosy prevalence rate in the area in 2009 was similar to the national rate of less than 0.5 case per 10,000 population there exists the presence of high and low endemic communities. Eight of the 32 Local Government Areas (LGA) making up the study states registered prevalence rates of more than 1 case per 10,000 population in 2009 [19], similar to new case detection rate (registered incidence). The two states have DG2 cases and child proportion higher than the national targets of 5% among new cases [8]. In Gombe state alone child proportion was 13.4% in the same period [19] indicating possible active transmission of leprosy in these communities.

### Study design

A cross-sectional study was designed across the high- and low-endemic communities of the states to evaluate a one year operational cost-effectiveness of the different case detection methods based on retrospective data available from 2005. The communities represented by the 32 LGAs were first categorised as either high-endemic or low-endemic using prevalence data from the NTBLCP 2009. Communities with registered prevalence rate of more than 1 case per10,000 population were categorised as high-endemic while those with less than 1 case per 10,000 population were categorised as low-endemic. Five communities were randomly selected from each endemic area. The ten sampled communities have a combined population of about 2,036,400. Selection was carried out to ensure geographical balance across the study area. Other criteria for selection were access to clinics providing MDT services for confirmed leprosy cases, data availability and comparable socio-economic status. Data was collected between 2005 and 2011. Effectiveness was measured as the number of new cases detected over a one year period and outcome expressed as cost per case detected.

### Intervention strategies assessed

Case detection or finding methods are categorised as passive and active methods. The passive case detection method, which constitutes routine practice in this study generally involves voluntary or self reporting by patients with suspected cases, and the active case finding methods at which health personnel visit patients at homes (contact tracing) or vicinities. Each of the detection strategies was implemented as a complement to routine method. The following methods were evaluated and compared.

### Passive case detection (PCD)

This represents the routine leprosy case detection method which integrates leprosy detection and control into the general health care system. It involves self-referral or referral of suspected cases by local health workers to leprosy units or peripheral health centre for examination by specialised health workers. The method involves the engagement of healthcare workers for the provision of leprosy services, regular staff training and supervision by state and local government Tuberculosis and Leprosy Staff (LGTBLS and STBLS), visits to health facilities (voluntary reporting) by patients, social mobilisation and health education.

### Household Contact Examination (HCE)

For our study contacts of index cases who share the same house and a kitchen are examined, and does not include neighbours and social contacts. It is characterised by the following activities;

- One-day orientation for health workers on contact examination
- Provision of N500 ($3) for each contact of an index patient examined, as transport
- Provision of guidelines on contact examination for general health workers to enhance implementation
- Listing of all index cases in the last 5 years from registers or existing records
- Examination of household members of index cases of leprosy, with expected average of 5 members based on expected family sizes in the study area
- Provision of general information on skin diseases for the household using message tools such as leaflets and providing contact details of health facilities for appropriate attention.

### Rapid Village Survey (RVS)

Key activities in RVS include;

- Identification of villages with leprosy cases in the last 5–7 years, with additional indicators for high child proportions and disability grade 2.
- Education campaigns on skin diseases and leprosy in selected villages targeting the population and key persons such as elders, teachers, religious leaders, and voluntary staff. Messages are delivered through leaflets and other communication media.
- Examination of persons voluntarily reporting.
- Routine programme case finding after the RVS
- Sometimes contact tracing of some individual cases are carried out

### Traditional healers' referral (THs)

Practitioners were identified through the Traditional Healers Association.whicu is recognised by government. They were trained to identify and refer suspected patients (based on presentation of skin conditions) to recognised centers for leprosy diagnosis. Two types of financial incentives were provided as motivation for the services. N50 ($0.33) was paid for each referred suspected case in the first instance while N500 ($3.30) was paid when leprosy was confirmed. The initial lower amount was paid as an incentive to encourage them to refer suspected skin conditions. It was made minimal to discourage abuse by excessive referrals. Activity summary include

- A consensus meeting with the Board of Traditional Healers (NATHP), to explore options for collaborations concerning leprosy case detection; offer of incentive for referrals of suspects and confirmation
- Selection and training of participants for the implementation
- One-day orientation for signs and symptoms of leprosy and where to refer suspects
- Orientation and courtesy visits to LGTBLS
- Provision of signed referral cards to be given to each suspect to referred health facility
- A feedback card to referred suspect to take back to the traditional healer to know the real status of the suspect
- Results compiled quarterly in the local government area (LGA)
- Process monitored by the LGTBLS and the state team.

### Ethical considerations

The study was carried out as part of routine leprosy control programme with no major contact with patients, requiring no ethical clearance. However the rights of the clients to supply information on family contacts to be examined were respected based on signed consent notes by the index patients. Agreement to participate in the questionnaire survey to estimate patients cost of seeking care was considered as consent.

### Measuring costs

Cost data were collected from expenditure records and reports. The costs of implementing services in all three methods were identified and measured using the ingredient approach, complemented by activity-based data. Bottom-up approach was used to estimate the economic costs, (where information on resource use and costs were available [20]) which involved identification and valuation of all resources required in the detection of new leprosy cases from the provider's perspective, as well as from the patient and family perspectives. Where detailed information was not available, top-down calculations was performed [21]. Table 1 presents sources of cost data and the method of estimation. Resources were first classified as capital and recurrent costs. Capital costs, which are used up beyond one year were obtained by annualization of the capital items over their expected life-span. All costs were converted to the US Dollar ($) at the 2010 exchange rate, ($1 = N152).

**Table 1:** Calculation methods and sources of data.

*Cost category*	*Calculation method*	*Data source*
Personnel	Top down	Salary records
Training/workshop	Top down	Accounts records
Social mobilisation	Top down	Programme/Accounts records
Incentives	Bottom up	Programme records
Transport	Bottom up	Accounts records
Shared costs	Top down	Staff interviews/programme records
Patients/family costs	Bottom-up	Questionnaire survey

Major areas of resource use include personnel, training/workshops, social mobilisation, transport, incentives. Personnel costs (staff salary and allowances) were based on the proportion of health worker/staff time devoted to leprosy case detection and allowances paid in the process, and these include doctors, nurses, healthcare workers etc. Salary data was collected from standard Nigerian payroll scale. Training and workshops comprised of short-term (recurrent) and long-term (capital) trainings and workshop costs. Costs of social mobilisation included such items as advocacy visits, Information, Education and Communication (IEC) materials, radio/TV adverts and promotion. Capital items included vehicles, motorcycles, long-term training and start-up costs. They were annualised over their useful time periods and discounted at 3% rate based on World Bank recommendation, capturing their depreciated costs as opportunity costs of time.

The routine programme costs consisted mainly of personnel, training and workshop (short and long-term), social mobilisation, vehicles and patient/family costs. The main cost elements for the RVS were allowances paid to field staff, training and workshop, social mobilisation and incentives provided in the form of de-worming agents given out to encourage attendance. No patient/family cost was recorded for RVS and HCE methods except as part of routine practice. HCE generated costs mainly from transportation and long-term training of field workers. Major cost elements for the THs approach were start-up expenditures (such as advocacy visits and mobilisation), long-term training for the traditional healers, allowances paid to field staff and the incentives paid out for referred cases by the traditional healers. However accuracy of these allocations was subject to availability of reliable data because resource use documentation was not detailed and properly defined (non-specific). Greater efforts were made at identifying and separating cost items for allocation to appropriate categories for analysis.

From patient and family perspective, costs incurred by the patients at the household were collected from a structured interview which targeted 50 outpatients from hospital/health facilities in the area of study. These costs included direct out-of-pocket expenses incurred in transportation and hospital/diagnostic fees (including multiple visits to obtain diagnosis). These costs include indirect costs of travel time and hospital (waiting) time. Hospital fees were not charged for leprosy at the time (data not available). The cost of time (time loss) was based on the prevailing minimum subsistence wage rate in Nigeria during the period of analysis. Newly approved rate was however used in sensitivity analysis to assess the impact on the study results.

### Shared costs

Most of the case finding activities particularly for HCE and RVS methods involved field activities which included the simultaneous provision of TB services. Costs incurred in the process were treated as shared costs. Consequently cost data for many services were adjusted for leprosy programme at proportions that reflected the level of resource use for leprosy case detection. For instance 5% of the cost of training general health care workers (GHCW) was assumed for leprosy case detection based on interviews with the workers. Similarly 30% and 40% personnel costs were estimated for state and local government controllers and supervisors respectively for case detection based on shared activities with TB and other leprosy services. Step-down approach was used to measure and allocate shared costs. Major proportions were however varied in a sensitivity analysis to explore the impact on the result.

### Start-up costs

Some cost items are generated at the beginning of some activities which are one-off and therefore expected to last for longer than one year over the life of the project. Such expenditures include trainings and the purchase of new equipments.

### Measuring effectiveness

Effectiveness data was measured in terms of number of newly confirmed leprosy cases detected within the period. These comprise of all patients diagnosed and confirmed with either PB or MB leprosy including child cases and disability grades 1 and 2 cases.

### Data analysis

All the data were tabulated and analysed using the Excel spreadsheet (Version 2007). Costing, worksheets were first created to collect relevant items for each method. The sheets contain the lists of likely resources used by each method. Data from the worksheets were then entered into the spreadsheet already programmed to calculate the required programme costs based on standard methods.

### Cost-Effectiveness Analysis

Comparison of costs and effects was based on incremental cost-effectiveness ratio (ICER). ICER seeks to identify an alternative that replaces an existing practice in the form of mutually exclusive option. It measures the additional cost that would be required to achieve more superior benefits (case detection) than the baseline [22]. This is defined as additional costs divided by additional benefits. This means that the differences in programme costs between each of the methods and routine case detection will be calculated and compared with differences in the new cases detected between each of the methods and routine PCD, to determine the ICER.

Thus:
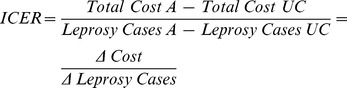
Where ‘A’ stands for alternative method (in this case UC+RVS, UC+HCE or UC+THs) and UC stands for Usual Care (PCD, the routine practice).

The method that yields the lowest ICER value is considered the most cost-effective alternative.

Hence the study used the ICER criteria to identify the most cost-effective method to replace existing practice for leprosy case detection.

However using average measure (average cost-effectiveness ratio, ACER) would mean that the method that produces the highest number of outcomes at a given/constant cost generates the lowest ACER, making it the most cost-effective and preferred option [22]. This is defined as total implementation costs divided by the total benefits. Using the average measure becomes relevant only when there is no existing practice and comparison between alternatives will be based on the ACER.

The ACER values are however presented for this study for complementary and comparative analysis.

### Sensitivity analyses

Some parameters that showed certain level of variability with potentials of affecting the study results were subjected to one-way sensitivity analysis to assess their impact and determine the robustness of the results. They include the discount rate, personnel costs, allocation factors for shared costs, subsistent (minimum) wage. Personnel costs were included because they constituted a major cost component. The cost-effectiveness values were recalculated using different values of these parameters in the sensitivity analysis.

## Results

### Effectiveness estimates/new leprosy cases detected

Table 2 summarises new leprosy cases detected by each method. From routine practice, an annual average of 164 cases was detected between 2005 and 2009.This was made up of 10.4% child cases and about 4.9% DG2. There were 138 cases in endemic areas and 26 cases in non-endemic areas. The RVS within the same period generated an annual average of 182 new cases with about 9.3% child cases and 6.0% DG2. For the HCE and THs method which were assessed between 2010 and 2011, 181 and 187 cases were detected respectively. As a major indicator for evaluating case detection and monitoring disease control progress [7], [9], the active detection methods identified more DG2 cases among the new cases than the routine passive method. The THs strategy detected the highest proportion of DG2 at about 8.6%. On the other hand the proportion of child cases, a indicator of leprosy disease transmission [23] was most detected by the routine detection method at 12.3%. MB cases accounted for over 82% of all the new leprosy cases.

**Table 2:** Distribution of annual cases by strategy.

*Detection Method*	*Cases detected*	*High-Endemic*	*Low-Endemic*	*PB*	*MB*	*DG2*	*Child cases*
**PCD**	164	138	26	29	135	8	17
**HCE**	181	153	28	30	151	14	17
**THs**	187	158	29	32	155	16	20
**RVS**	182	153	29	32	151	11	17

### Programme costs

The summary of the annual programme costs estimated for each method is shown in Table 3 by category, presented for both the provider and patients/family perspectives. It shows for each method, the relative composition of resource input for detection of one leprosy case in the study area for a period of one year. The RVS strategy was shown to produce the highest cost input per patient, at about $210 per case. Personnel cost was the largest single item in all methods accounting for 57–65% of total costs, apparently due to high personnel requirements, especially for routine practice. It accounted for 65% of routine practice cost which recorded $198 per case. Training and workshop as well as social mobilisation were the other major cost items generating 10–21% of total resource inputs among the strategies. The high cost of social mobilisation was also due to the number of activities involved in mobilising communities for leprosy detection such as advocacies, education, communications etc. At $193 per case detected the HCE produced the lowest unit cost among the strategies with personnel responsible for 61%. Transport made the most significant impact at 9.1% of HCE programme cost. THs produced the second lowest unit cost per case at about $197. Interestingly incentive was not a significant item in the THs method.

**Table 3:** Programme unit costs (US$).

*Perspective*	*Cost category*	*Item*	*PCD (UC)*	*UC + RVS*	*UC + HCE*	*UC + THs*
***Provider***	*Recurrent*	Personnel	128.2	118.9	116.6	113.5
		Training/Workshop	21.8	43.8	19.8	19.1
		Social Mobilisation	35.4	32.3	32.1	31.2
		Transport	5.3	5.1	9.1	4.6
		Material supplies	1.4	1.3	1.6	1.4
		Rentals	0.0	0.2	0.0	0.0
		Incentives	0.0	2.8	0.0	4.8
	Capital Cost	Start-up costs	0.0	0.0	0.0	3.8
		Training; long-term	0.3	0.3	8.6	14.0
		Vehicles	3.0	2.7	2.8	2.7
		***Subtotal***	***195.6***	***207.4***	***190.5***	***195.1***
***Patient & Family***	Direct	Transportation	1.3	1.2	1.2	1.3
		Hospital fees	0.0	0.0	0.0	0.0
	Indirect	Hospital time	0.2	0.2	0.2	0.2
		Travel time	0.9	0.8	0.8	0.8
		***Subtotal***	***2.4***	***2.2***	***2.2***	***2.3***
		***Total (US$)***	***198.0***	***209.6***	***192.7***	***197.4***

Patient/family costs contributed only about 1% of the total programme cost per patient, generated only by routine practice and the THs strategies. Average transportation costs to and from health care facility was $1.32 per patient while on the average a patient visits healthcare facilities 1.5 times before being diagnosed for leprosy, ranging from 1 to 4 times. Average travel and waiting times totalled 92 minutes (61 min and 31 min respectively), which translates to approximately $0.41 per patient based on the minimum subsistent wage rate during the period of study. New minimum wage rate was used to assess the impact in sensitivity analysis.

### Incremental Cost-Effectiveness Ratios (ICER)

The respective ICERs for the different options are presented in Table 4. It is presented for providers/patients perspectives at all contact levels, to guide appropriate implementation decisions. The ICER indicates the cost (in US dollars) for each additional leprosy case. For the HCE plus usual care strategy, additional $2,416 was expended to detect 17 additional cases during the period, resulting to $142 per additional leprosy case detected. It was most cost-effective in high- endemic areas at $81 per additional case detected than in low-endemic areas where it produced a total of $604 per additional case detected. The HCE was closely followed by the THs plus usual case strategy which produced additional 23 leprosy cases at a total cost of $4,447 to yield $194 per additional case detected. Results show that RVS was not cost-effective given by the dominance of the THs method which generated lower cost and higher effectiveness than the RVS. Findings were similar from both provider's and patient's' perspectives and at different contact levels. At both high and low-endemic areas, the HCE generated the lowest ICERs, making it the most cost-effective method. The RVS method was similarly not cost-effective in those areas as it was again dominated by the THs method. Average values produced similar results which show the HCE having the lowest ACER (Table 4).

**Table 4:** Cost-Effectiveness Estimates.

Detection method	Cost (N)	Cases detected	Incremental cost (N)	Incremental cases	ICER (N)	*ICER (US$)*	ACER ($)
**PCD**	4,935,325	164	0	0	0	*0*	198
**HCE**	5,302,501	181	367,176	17	21,599	*142*	193
**THs**	5,612,274	187	676,949	23	29,433	*194*	197
**RVS**	5,798,879	182	863,554	18	*47,975*	*316*	210

### Sensitivity analysis

Results of varying major parameters on the CERs are presented in Table 5 which showed that personnel costs, shared costs proportions, subsistence wage, discount rate and diagnostic accuracy did not significantly alter study results although impact on programme costs were significant for changes in the shared costs proportions and personnel costs. When the shared costs proportions for leprosy detection were increased by 50%, programme costs for each strategy were increased by an average of 40% but the ICERs did not change. When the proportion was reduced by 50%, programme costs reduced by an average of 27% across the methods, with little or no change in the ICER results. However the cost-effectiveness of the RVS significantly improved by 27%, from being dominated to $221 per additional case detected. When the subsistent wage was increased from N7,000 to N18,900 in view of the current wage structure, the impact on the study results was insignificant. When the personnel cost was increased by 30% only the programme costs increased by about 18%, with no impact on the ICER results.

**Table 5:** Results of sensitivity analysis of selected parameters.

*Parameters of interest analyzed*	*Scenarios evaluated*	*%Change in parameters*	*PCD*	*RVS*	*HCE*	*THs*
**Shared costs**	50%	Program Cost (%)	43	40	40	38
		***ICER (%)***	0	0	0	0
	−50%	Program Cost (%)	−29	−29	−27	−25
		***ICER (%)***	0	−29%	0	0
**Minimum wage/salary**						
	18,900	Program Cost (%)	19	16	18	17
		***ICER (%)***	0	0	0	0
**Diagnostic accuracy**	Not available					

## Discussion

This cost-effectiveness study is the first of its kind in leprosy control studies as no report of this nature, to our knowledge has been documented in the recent past. The study was an operational cost-effectiveness analysis of leprosy case detection methods (in routine practice), relying mainly on retrospective data between 2005 and 2011. It is based on integration of each strategy with routine method to complement and improve existing practice in detection of new leprosy cases. The study indicated that HCE, in its present definition is the most cost-effective alternative in detecting new leprosy cases in the study area, generating $142 per additional case detected in relation to routine practice alone. The robustness of this finding was evidenced in all contact levels and from both providers' as well as patients/family perspectives which showed similar results. The perspectives of the study were necessary to analyse the costs and benefits data from broader viewpoints that include the patients/family for a more balanced and comprehensive decisions, such as considering the need for subsidy. Comparative effectiveness within the study period suggested that the active detection methods were more effective at detecting DG2 cases than the passive routine method, with the THs strategy having the highest proportion of DG2 among the new cases. This is not surprising since due to stigma associated with leprosy the patients are less likely to present at public health facilities. Poor access to public health facilities may also contribute to low case detection rate in the routine method.

Since no study has been documented on the cost-effectiveness of case detection methods, it was not possible to make comparisons. Published studies on cost-effectiveness analysis of leprosy interventions are very limited [4], and none is related to case detection, making comparison of study findings difficult. At $142 per additional case detected, it compares favourably with other leprosy interventions. A study in Bangladesh [24] evaluated the cost-effectiveness of chemo-prophylactic intervention with a single dose Rifampicin in household contacts of new leprosy patients, which resulted in a CER of $158 per additional case prevented. The study presented an interesting finding on the cost-effectiveness of chemoprophylaxis with rifampicin, implemented in conjunction with HCE. It demonstrated greater cost-effectiveness of leprosy prevention. Although the addition of rifampicin represents a limited additional cost to the intervention, it added significantly to the effect of the HCE. Applying this approach to the HCE as a routine strategy in the Nigerian context will further reinforce the cost-effectiveness of the strategy in leprosy case detection.

The CER falls within the WHO's category of studies classified as highly cost-effective interventions, being less than three times the Gross Domestic Product (GDP).The low unit cost per case detected at $193 indicates that resource use is lower or more efficient in detection of more cases, suggesting that it will be easier to scale-up to achieve increased case detection rates to enhance elimination of leprosy.

The study justifies the use of HCE as a routine method in many countries in line with WHO recommendation [11]. Many factors can explain the findings, such as implementation costs which was the lowest per unit of output (per case detected). Leveraging on existing health care facility, the method requires less manpower. Results of sensitivity analysis reinforce HCE as the most cost-effective method in the study area, providing insight into the likely changes in the study findings in other settings, further reinforcing the findings. However it is noteworthy that the cost-effectiveness of the THs strategy is close to the HCE in all cases. The method also detected most DG2 cases compared to other strategies and this is instructive for achieving the objective of reducing the DG2 incidence as a major focus of the National Strategic Plan. This suggests that implementation of the THs strategy in combination with HCE, especially in areas where it was found to be most cost-effective will maximise case detection and help identify more of the DG2 cases. Similarly the RVS was found to be most cost-effective in Adamawa state and reducing the leprosy proportion of shared cost increased the overall cost-effectiveness of the strategy. This suggests that smarter use of resources to reduce cost without compromising quality will increase the cost-effectiveness of the RVS and targeted combination of strategies will generally enhance the efficiency of the detection methods.

### Limitations of the study

Interpretations of the result need to be carried out in relation to some limitations of the study which is important for generalising the findings. Based on retrospective data from routine practice, it is subject to recall biases. Resource use data were not very detailed and specific, resulting in greater efforts at separating and allocating resources which, in many cases were arbitrary. The gap in data availability may have resulted in some costs not adequately captured. Hence cost allocations may not be very appropriate and accurate requiring the need for field implementation to obtain more accurate results, by correcting identified challenges. This was general for all methods.

Diagnostic accuracy was also not very specific as separation of cases between the PCD and RVS methods was in some cases challenging due to poor data documentation, which again would be addressed in a field study. Analysis of effectiveness data did not consider future impact beyond a one year period as the mix of leprosy cases detected have varying benefits from preventing the progression to permanent disability. The higher the number of early disease cases the greater the potential benefits of being prevented from progression to permanent disability, with MDT treatment. The use of DALY as a measure of outcome will involve the measure of utility for each leprosy grade detected such that the higher the number of early cases the higher the utility and hence the benefits. Lack of data on utility values and inadequate information on the proportion or distribution of leprosy categories limited this approach. However this may not have changed the study findings since the methods yielded similar proportions of child and DG cases. Definition of HCE in the study was the narrowest, limiting contact of index cases to household members, excluding neighbours and neighbours of neighbours which have been shown to increase case detection [23], [24]. Hence broader definition of contact tracing would increase the cost-effectiveness of HCE. Lastly given the dynamic nature of leprosy transmission as a communicable disease, the study did not capture the benefits of prevention beyond one year, but was only based on analysis of primary prevention. A dynamic model would have captured the benefits of further prevention beyond the index cases but this was not possible as information on the long term impact of prevention is not available. This would also have increased the cost-effectiveness of the various methods, especially for HCE.

In addition to the study limitations it would also be necessary to consider other factors such as economic differences between countries when generalising the study results. Programme costs will likely differ due to varying price levels of items such as personnel salaries and allowances, etc, leading to possible variation in cost-effectiveness results.

### Recommendations

In view of the findings from the study we make the following recommendations to enhance effective detection and control of leprosy in Nigeria.

1. The study findings suggest the need to carefully implement and integrate HCE into routine practice for more effective identification and control of leprosy in the area. Furthermore broadening the definition of contact tracing to include neighbours of neighbours would increase the rate of detection, since the cost-effectiveness in the study was achieved at the narrowest definition of contact tracing. With established benefits with chemoprophylactic combination with rifampicin, the cost-effectiveness of the HCE would be further enhanced.
2. The lower cost of implementation of the HCE which generated the lowest average cost per case detected (ACER) offers the best option for scale-up to increase coverage and the detection of more leprosy cases.
3. The Traditional Healers incentive method demonstrated value close to the HCE as the next most cost-effective strategy. This suggests the need for a combination of the two strategies for a more comprehensive case detection, to cover potential cases that often rely on traditional healers for healthcare service, and enhance elimination of leprosy. It is also possible to implement the RVS method as part of the integration, in areas where it was most cost-effective. These combinations would maximise the benefits of each method without affecting the budget.
4. In recognition of the many limitations of the study related to data collection due to the retrospective perspective of routine practice, it would be best to carry out implementation studies in the field to correct data gaps and improve accuracy and reliability of study findings.

### Conclusion

The study has shown that the Household Contact Examination is the most cost-effective approach for identifying new leprosy cases when implemented to complement routine practice in Nigeria. It offers the best option for scale-up for improved coverage having the lowest average cost per case detected. Broadening the definition of contact tracing will enhance detection of more leprosy cases for improved control. The combination of HCE with chemoprophylaxis would further enhance the cost-effectiveness of the intervention. Integration of the strategy into routine practice is therefore recommended for improved case detection of leprosy in Nigeria, depending on its acceptability and feasibility in the area. The finding also justifies the WHO recommendation for inclusion of contact tracing into routine case detection. However due to challenges in data collection (and generally in cost-effectiveness studies), large differences in results make more sense than the little differences recorded in this study. Hence the cost-effectiveness of the three methods can be considered similar and implementation of the methods in a budget neutral way may be necessary to maximise the detection of leprosy in the area, targeting specific areas where each method is considered more effective.
